# Rapid evolution of metabolic traits explains thermal adaptation in phytoplankton

**DOI:** 10.1111/ele.12545

**Published:** 2015-11-26

**Authors:** Daniel Padfield, Genevieve Yvon‐Durocher, Angus Buckling, Simon Jennings, Gabriel Yvon‐Durocher

**Affiliations:** ^1^ Environment and Sustainability InstituteUniversity of ExeterPenrynCornwallTR10 9FEUK; ^2^ School of Biological and Chemical SciencesQueen Mary University of LondonLondonE1 4NSUK; ^3^ Centre for EnvironmentFisheries and Aquaculture ScienceLowestoftNR33 0HTUK; ^4^ School of Environmental SciencesUniversity of East AngliaNorwichNR4 7TJUK

**Keywords:** Carbon cycle, metabolic theory, phytoplankton, rapid evolution

## Abstract

Understanding the mechanisms that determine how phytoplankton adapt to warming will substantially improve the realism of models describing ecological and biogeochemical effects of climate change. Here, we quantify the evolution of elevated thermal tolerance in the phytoplankton, *Chlorella vulgaris*. Initially, population growth was limited at higher temperatures because respiration was more sensitive to temperature than photosynthesis meaning less carbon was available for growth. Tolerance to high temperature evolved after ≈ 100 generations via greater down‐regulation of respiration relative to photosynthesis. By down‐regulating respiration, phytoplankton overcame the metabolic constraint imposed by the greater temperature sensitivity of respiration and more efficiently allocated fixed carbon to growth. Rapid evolution of carbon‐use efficiency provides a potentially general mechanism for thermal adaptation in phytoplankton and implies that evolutionary responses in phytoplankton will modify biogeochemical cycles and hence food web structure and function under warming. Models of climate futures that ignore adaptation would usefully be revisited.

## Introduction

Phytoplankton play a key role in biogeochemical cycles (Field 1998) and fuel aquatic food webs (Falkowski *et al*. 2008). Novel phytoplankton communities, and the functions they mediate, will emerge as the climate changes through a combination of turnover in species composition (Yvon‐Durocher *et al*. 2011), and shifts in the distribution of traits (e.g. body size, metabolic rates, stoichiometry) via phenotypic plasticity (Schaum *et al*. 2013; Magozzi & Calosi 2014) and rapid evolution (Lohbeck *et al*. 2012; Schaum & Collins 2014; Schlüter *et al*. 2014; Geerts *et al*. 2015). The amount of plasticity and evolutionary potential in key traits, relative to their interspecific variability (Thomas *et al*. 2012), will largely determine the extent to which phytoplankton communities are buffered from species turnover in a warmer world (Pörtner & Farrell 2008; Angilletta 2009; Montoya & Raffaelli 2010).

Because of their rapid generation times and high population densities, phytoplankton have substantial capacity for rapid evolutionary responses to climate change (Collins 2011). There is growing evidence for evolutionary responses of phytoplankton *in vitro* to global change drivers, such as elevated CO_2_ (Collins & Bell 2004; Lohbeck *et al*. 2012; Schaum & Collins 2014; Schlüter *et al*. 2014), but only a single study has explored responses to warming (Schlüter *et al*. 2014). Studies applying experimental evolution to phytoplankton have focused mainly on identifying the capacity for adaptation and have not investigated the underlying mechanisms that facilitate evolutionary responses. Understanding the capacity for, and mechanisms through which phytoplankton might evolve to cope with novel environments is central to predicting whether aquatic ecosystems will accelerate or mitigate global warming through changes in their capacity to sequester carbon. Such quantitative and mechanistic understanding will support the development of more realistic models of the ecological and biogeochemical effects of climate futures.

Thermal tolerance – the range of temperatures at which an organism can grow – is expected to be critical for determining species' responses to global warming (Pörtner & Farrell 2008; Kearney *et al*. 2009). Evolution has driven substantial variation in thermal tolerance among species of phytoplankton adapted to different environments (Thomas *et al*. 2012). Experiments on bacteria (Bennett & Lenski 2007), evolving and coevolving viruses (Zhang & Buckling 2011) and zooplankton (Geerts *et al*. 2015) have demonstrated capacity for rapid evolution of elevated thermal tolerance. However, the extent, tempo and mechanisms through which elevated thermal tolerance can evolve in phytoplankton are currently unclear.

**Figure 1:**
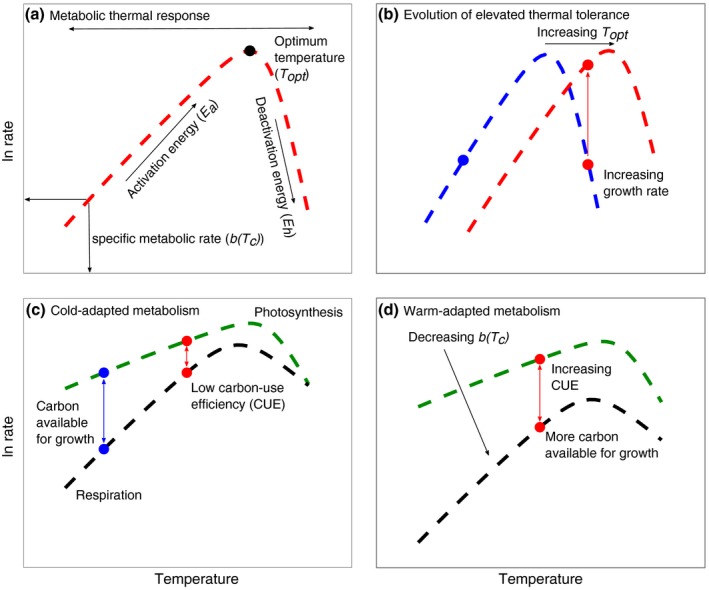
Effects of temperature on phytoplankton metabolism. (a) Metabolic rates, *b*(*T*), increase as an exponential function of temperature to an ‘optimum’ temperature (*T*_opt_), then decline rapidly. (b) Growth responses for a cold‐adapted (blue, point & line) and warm‐adapted (red, point & line) phytoplankton. Evolution of elevated thermal tolerance entails increases in *T*_opt_ and growth rates at elevated temperatures. (c) Thermal responses for photosynthesis (green, *P*) and respiration (black, *R*) for a cold‐adapted phytoplankton. The relative difference between *P* and *R* along the temperature gradient represents the carbon use efficiency (CUE = 1−*R/P*). Because *R* is more sensitive to temperature than *P*, CUE declines (blue to red point) with increasing temperature, limiting growth at high temperatures. As phytoplankton adapt to warmer temperature (d), selection should drive down specific rates of respiration, *R*(*T*_*c*_), more than those of photosynthesis, *P*(*T*_*c*_), increasing CUE and ensuring they are not limiting for growth.

Metabolism sets the pace of life (Brown *et al*. 2004) and is a key process that can be used to gain a more mechanistic understanding of evolutionary responses to changes in temperature. Metabolism dictates a host of life‐history traits and attributes that determine fitness, including population growth rate (Savage *et al*. 2004), abundance, mortality and interspecific interactions (Dell *et al*. 2011). During acute exposure to a range of temperatures, metabolic rates, *b*(*T*), typically increase exponentially up to an optimum, followed by a rapid decline (Fig. 1a). These unimodal thermal response curves can be described using a modification of the Sharpe–Schoolfield equation for high temperature inactivation (Schoolfield *et al*. 1981):

(1) $$\text{ln}\left(b\left(T\right)\right)=E_{a}\left(\frac{1}{kT_{c}}-\frac{1}{kT}\right)+\text{ln}\left(b\left(T_{c}\right)\right)-\text{ln}\left(1+e^{E_{h}\left(\frac{1}{kT_{h}}-\frac{1}{kT}\right)}\right)$$

where *b*(*T*), is the metabolic rate per unit biomass (μmol O_2_ μg C^−1 ^h^−1^), *k* is Boltzmann's constant (8.62 × 10^−5^ eV K^−1^), *E*_*a*_ is an activation energy (in electron volts, eV) for the metabolic process, *T* is temperature in Kelvin (K), *E*_*h*_ characterises temperature‐induced inactivation of enzyme kinetics above *T*_*h*_ and *b*(*T*_*c*_) is the rate of metabolism normalised to a reference temperature, *T*_*c*_ = 25 °C ; where no low or high temperature inactivation is experienced (we refer to *b(T_c_)* as the ‘specific rate of metabolism’). Because *b*(*T*_*c*_), is both mass and temperature normalised it enables comparison of metabolic rates across populations which may vary in total biomass and/or ambient temperature. Equation (1) yields a maximum metabolic rate at an optimum temperature:

(2) $$T_{\mathrm{opt}}=\frac{E_{h}T_{h}}{E_{h}+kT_{h}\text{ln}\left(\frac{E_{h}}{E_{a}}-1\right)}$$

the parameters *b*(*T*_*c*_), *E*_*a*_, *E*_*h*_, and *T*_opt_, represent metabolic traits that together characterise the metabolic thermal response (see Fig. 1a)

In phytoplankton, growth rate (a component of fitness) is dependent on two metabolic fluxes: photosynthesis and respiration (Raven & Geider 1988). We hypothesise that selection will operate on the metabolic traits of these two fluxes as species adapt to new thermal environments. Past work suggests that respiration (activation energy for respiration: *E*_*R*_ **~ 0.65 eV) is often more sensitive to temperature than photosynthesis (*E*_*P*_ ~ 0.32 eV) (Allen *et al*. 2005; López‐Urrutia *et al*. 2006; Anderson‐Teixeira *et al*. 2011; Yvon‐Durocher *et al*. 2012). As temperatures rise, rates of respiration rise relatively more than those of photosynthesis. Consequently, the ratio of respiration (*R*; i.e., the gross remineralisation of organic carbon (C)) to gross photosynthesis (*P*; i.e., the gross fixation of inorganic C), *R*/*P*, increases as a function of temperature, which means the fraction of C available for growth after satisfying the catabolic demands of the cell, termed carbon‐use efficiency (CUE = 1−*R*/*P*), declines as temperatures rise in the short term (Gifford 2003; Allison *et al*. 2010) (Fig. 1c). This poses a major physiological challenge for photoautotrophic growth at high temperatures. We hypothesise that adaptation to warmer temperatures (Fig. 1b) should arise via evolutionary adjustments to metabolic traits that serve to increase CUE (Allison 2014) and partially offset intrinsic declines in CUE driven by the differences in the temperature sensitivity of *R* and *P* (Fig. 1d). When the activation energy for *R* is greater than *P* (*E_R_* > *E_P_*), specific rates of respiration, *R*(*T_c_*), should decline more than those of photosynthesis, *P*(*T_c_*), resulting in increases in the specific carbon‐use efficiency [CUE(*T_c_*) = 1‐*R(T_c_)*/*P(T_c_)*] as phytoplankton adapt to higher temperatures (Fig. 1d). CUE(*T_c_*) reflects the carbon‐use efficiency normalised to the reference temperature *T_c_* = 25°C and controls for the intrinsic temperature responses of *R* and *P*. Here, we test these hypotheses by combining experimental evolution (Buckling *et al*. 2009; Reusch & Boyd 2013) with measurements of fundamental physiology to investigate mechanisms of thermal adaptation in the model freshwater alga, *Chlorella vulgaris*.

## Methods

### Culture conditions

*Chlorella vulgaris* is a globally distributed alga and has been found across North and South America, Asia, Europe and Australasia (Algaebase 2015). The particular strain (A60 strain, Sciento) used here was isolated from a pond in northern England 15 years ago and has since been maintained in laboratory culture at 20 °C. Three replicate populations of the A60 strain of *C. vulgaris* were established at five different temperatures and were grown under nutrient and light saturated conditions in Infors‐HT shaking incubators (160 rpm) on a 12 : 12 light:dark cycle and with a light intensity of 175 μmol^−1 ^m^−2^ s^−1^. Cultures were grown in 200 mL of Bold's Basal Medium, supplemented with NaHCO_3_ (0.0095 M). These conditions represent typical benign conditions for this strain. Note that we initiated our experimental treatments with populations that presumably contained pre‐existing genotypic variation, rather than single clones, to maximise the response to selection and better reflect evolutionary responses expected from natural populations (Reusch & Boyd 2013).

**Figure 2:**
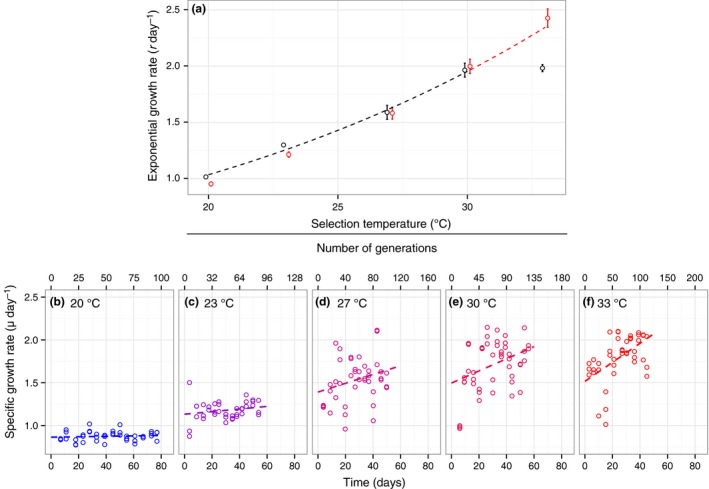
Growth rate trajectories for *Chlorella vulgaris* at different selection temperatures. (a) Exponential rates of population growth (*r*) at the five different selection temperatures after 10 generations (black circles) and 100 generations (red circles). The broken black line shows the temperature dependence of growth rate for the all lineages up to 30 °C after 10 generations and the broken red line predicts the expected growth rate at 33 °C from these data fitted to the Boltzmann–Arrhenius model (see Methods). (b–f) Trajectories of specific growth rate (μ) for populations at 20 °C (the ancestral temperature), 23, 27, 30 and 33 °C respectively. Broken lines in (b–f) show growth trends based on the fixed effects of a linear mixed effect model (see Methods).

Selection temperatures included the long‐term ancestral growth temperature of the strain, 20 °C, and 4 warming scenarios: 23, 27, 30 and 33 °C. Initial experiments indicated that 33 °C was beyond the optimal growth temperature (30 °C) and was therefore selected as the maximum experimental temperature to investigate the evolution of elevated thermal tolerance (see Fig. 2a). Exponential growth was maintained in semi‐continuous batch culture; during the mid‐log growth phase (identified from pilot growth experiments), 1 × 10^3^ cells were transferred into new media to prevent resource limitation. Physiology and growth curve measurements were made twice on each of the three biological replicates at each selection temperature after 10 and 100 generations. The absolute time taken to reach 100 generations varied from 45 to 77 days depending on selection temperature (see Fig. 2b–f).

### Growth rates

Population biomass (μg C mL^−1^) was measured at each transfer using a particle counter, which uses electrical sensing flow impedance determination to count and size cells (CellFacts^™^). Measurements of cell volume (μm^3^) were transformed into units of carbon (μg C cell^−1^) following Montagnes & Berges (1994). Specific growth rate, μ (d^−1^), was calculated as:

(3) $$\mu =\frac{\ln(N_{1}/N_{0})}{\Delta T}$$

where *N*_1_ is the final biomass (μg C), *N*_0_ represents the initial biomass and Δ*T* is the time interval (d). The number of generations per transfer (*g*) is equivalent to the number of doubles and was calculated as follows:

(4) $$g=\frac{\Delta T}{\ln\left(2\right)/\mu }$$

where Δ*T* is the time interval of the transfer (d), ln(2)/μ is the doubling time (d) and μ is the specific growth rate (d^−1^). We used linear mixed‐effects modelling to quantify trajectories in specific growth rate at the different selection temperatures, allowing us to control for the hierarchical structure of the data (e.g. variance at the replicate level nested within selection temperatures), heteroscedasticity and temporal autocorrelation (Pinheiro & Bates 2006). For the analysis, μ was the dependent variable, time (days) and selection temperature were fixed effects, while slopes and intercepts were treated as random effects at the level of replicates nested within selection temperatures (Table 1). We controlled for heteroscedasticity by modelling changes in variance with selection temperature, and temporal autocorrelation using an autoregressive moving average function at the level of the random effect. Significance of the parameters were assessed using likelihood ratio tests, comparing models with common slopes and intercepts to models with different slopes and intercepts for each selection temperature (Table 1). Multiple comparison tests using Tukey's least significant difference were used to determine pairwise parameter differences between selection temperatures and significant differences from 0 (Table S1). Model selection was carried out on models fitted using maximum likelihood, while multiple comparison tests were carried out on the most parsimonious model fitted using restricted maximum likelihood.

**Table 1:** Results of the linear mixed effects model analysis for trajectories of specific growth rate (μ; see Methods)

Model	d.f.	AIC	Log Lik	*L*‐ratio	*P*
Random effects structure					
random = ~ 1¦id					
corr. structure = varPower() & corARMA(q = 1)					
Fixed effects structure					
1. growth rate ~ 1 + time * selection temp	14	−140	84		
2. growth rate ~ 1 + time + selection temp	10	−123	71.3	25.4	< 0.001
3. growth rate ~ 1 + time	6	−53	32.9	77.0	< 0.001

Random effects on the slope and intercept were determined at the level of replicates nested within selection temperatures. The results of the model selection procedure on the fixed effect terms are given and the most parsimonious model is highlighted in bold. Analyses reveal that growth rates changed significantly through time and that growth trajectories were significantly different between selection temperatures.

Exponential population growth rates (*r* (d^−1^)) were calculated from logistic growth curves, measured after 10 and 100 generations at each selection temperature (Fig. 2a and Fig. S2). Samples were taken twice daily to estimate biomass, and once the stationary phase had been reached, we fitted the logistic growth equation to the biomass data using non‐linear least squares regression:

(5) $$N\left(t\right)=\frac{K}{1+Ae^{-rt}};A=\frac{K-N_{0}}{N_{0}}$$

where *N(t)* is the number of individuals at time, *t*,* K* is the carrying capacity, *N*_0_ is the number of individuals at the start of the sampling period and *r* is the rate of exponential growth.

### Characterising the metabolic thermal response

Responses of photosynthesis and respiration to acute temperature variation were determined across a broad range of temperatures (10 °C – 49 °C) to characterise the metabolic thermal response of *Chlorella vulgaris* (Fig. 1). 10 mL aliquots of the populations were concentrated through centrifugation and acclimatised to the assay temperature for 15 minutes in the dark before measuring metabolic rates. Photosynthesis and respiration were measured through oxygen evolution in the light, and oxygen consumption in the dark, on a Clark‐type oxygen electrode (Hansatech Ltd, King's Lynn UK Chlorolab2). Photosynthesis was measured at increasing light intensities in intervals of 30 μmol^−1 ^m^−2^ s^−1^ up to 300 μmol^−1 ^m^−2^ s^−1^, and then in intervals of 100 μmol^−1 ^m^−2^ s^−1^ up to 1000 μmol^−1 ^m^−2^ s^−1^. This yielded a photosynthesis irradiance curve (PI) at each assay temperature. PI curves were fitted to the photoinhibition model (Platt *et al*. 1990) using non‐linear least squares regression (Fig. S3):

(6) $$P\left(I\right)=P_{s}(1-e^{\frac{-\alpha I}{P_{s}}}e^{\frac{-\beta .I}{P_{s}}})$$

where *P*(*I*) is the rate of photosynthesis at light intensity, *I*,* P*_*s*_ is a scaling coefficient that sets the relative rate of *P*, α controls the rate at which *P*(*I*) increases up to a maximal rate, and β determines the extent to which *P* declines after the optimal light intensity due to photoinhibition. The photosynthetic maximum, *P*_max_, was then calculated from eqn (5) as:

(7) $$P_{\max}=\frac{P_{s}}{\left(\frac{\alpha +\beta }{\alpha }\right){\left(\frac{\beta }{\alpha +\beta }\right)}^{\frac{-\beta }{\alpha }}}$$

Rates of respiration were measured in the dark. Gross photosynthesis (*P*) was then estimated as *P *= *P*_max_ + *R*. By using *P*_max_ we can control for any potential light‐temperature interactions in the characterisation of the thermal response for *P*. Rates of *P* and *R* at each assay temperature were normalised by dividing by the biomass measured in each aliquot.

Acute responses of biomass normalised *P* and *R* to temperature were fitted to a modified Sharpe‐Schoolfield equation for high temperature inactivation (see eqn (1)) using non‐linear least squares regression. Fits were determined using the ‘nlsLM’ function in the ‘minpack.lm’ (Elzhov *et al*. 2009) package in R statistical software (v3.2.0) (R Core Team 2014), which uses the Levenberg‐Marquardt optimisation algorithm. Model selection using the Akaike Information Criterion (AIC) was carried out to identify the parameter set which best characterised the data. This entailed running 1000 different random combinations of starting parameters drawn from a uniform distribution and retaining the parameter set that returned the lowest AIC score. The goodness of fit of the selected models were examined both graphically and through computation of a pseudo‐*R* ^2^ value, recognising the caveats associated with calculating *R* ^2^ for non‐linear models (Spiess & Neumeyer 2010). We tested for the effects of ‘selection temperature’ on the metabolic traits (parameters of eqn (1) for *P* and *R*) using the Boltzmann–Arrhenius function:

(8) $$\text{ln}\left(b\left(T\right)\right)=E_{a}\left(\frac{1}{kT_{c}}-\frac{1}{kT}\right)+\text{ln}\left(b(T_{c}\right))$$

where *b*(*T*) is the metabolic trait at the selection temperature, *T*,* b*(*T*_*c*_) is the trait at temperature *T*_*c*_, where *T*_c_ = 25 °C, and *E*_*a*_ is the activation energy that determines how quickly the trait varies as a function of *T*. We used eqn (8) in an Analysis of Covariance to test for the effects of ‘selection temperature’, ‘exposure’ (e.g. long‐term vs. short‐term warming), and ‘metabolic flux’ (either *P* or *R*) on the parameter estimates (Table 2).

**Table 2:** Results of an Analysis of Covariance for each metabolic trait with interactions between ‘selection temperature’, ‘exposure’ (short‐ or long‐term warming) and ‘metabolic flux’ (*P* or *R*)

Parameter	Effect	d.f.	*F* value	*P* value
*b(T*_*c*_ *)*	**selection temperature**	1,52	13.7	**< 0.001**
	**metabolic flux**	1,52	274.58	**< 0.001**
	**exposure**	1,52	40.9	**< 0.001**
	selection temperature*metabolic flux	1,52	0.198	0.65
	selection temperature*exposure	1,52	6.93	**< 0.05**
	metabolic flux*exposure	1,52	2.38	0.13
	selection temperature*metabolic flux*exposure	1,52	1.36	0.25
*E*_*a*_	selection temperature	1,52	2.44	0.11
	**metabolic flux**	1,52	56.8	**< 0.001**
	**exposure**	1,52	7.29	**< 0.01**
	selection temperature*metabolic flux	1,52	0.0004	0.98
	selection temperature*exposure	1,52	0.024	0.88
	metabolic flux*exposure	1,52	4.8	**< 0.05**
	selection temperature*metabolic flux*exposure	1,52	0.91	0.34
*E*_*h*_	**selection temperature**	1,52	4.71	**< 0.05**
	**metabolic flux**	1,52	17.5	**< 0.001**
	exposure	1,52	1.78	0.19
	selection temperature*metabolic flux	1,52	0.42	0.52
	selection temperature*exposure	1,52	0.09	0.76
	metabolic flux*exposure	1,52	0.39	0.53
	selection temperature*metabolic flux*exposure	1,52	0.002	0.96
*T*_*h*_	selection temperature	1,52	0.15	0.34
	**metabolic flux**	1,52	36.4	**< 0.001**
	**exposure**	1,52	4.73	**< 0.05**
	selection temperature*metabolic flux	1,52	0.335	0.57
	selection temperature*exposure	1,52	0.211	0.65
	metabolic flux*exposure	1,52	1.38	0.25
	selection temperature*metabolic flux*exposure	1,52	0.23	0.64
*T*_opt_	selection temperature	1,52	2.23	0.133
	**metabolic flux**	1,52	28.8	**< 0.001**
	exposure	1,52	2.75	0.10
	selection temperature*metabolic flux	1,52	0.04	0.85
	selection temperature*exposure	1,52	0.02	0.88
	metabolic flux*exposure	1,52	5.81	**< 0.05**
	selection temperature*metabolic flux*exposure	1,52	0.0001	0.99

Parameters included in the most parsimonious model are highlighted in bold.

In plant physiology, the carbon‐use efficiency (CUE) represents the fraction of fixed carbon that is available for allocation to growth (Gifford 2003), and can be estimated from rates of gross photosynthesis (*P*) and respiration (*R*) as: CUE = 1−*R*/*P*. CUE and specific carbon‐use efficiency [CUE(*T_c_*)] were estimated for each replicate after 10 and 100 generations from rates of *R* and *P* measured at their selection temperature and from the specific rates, *R*(*T_c_*), and *P*(*T_c_*), respectively. Because our cultures experienced a 12 : 12 h light‐dark cycle, integrated over 24 h, populations will be photosynthesising for 12 h but respiring for 24 h. In estimating CUE, rates of *R* and *P* were integrated over 24 and 12 h respectively, to account for the diel population‐level carbon budget. We then analysed the estimates of CUE and CUE(*T_c_*) using Analysis of Covariance, where ‘selection temperature’ and ‘exposure’ (e.g. long‐term vs. short‐term warming) were included as potentially interacting factors. Selection temperature was centered (*T* – *T_c_*) so that the intercept of the linear model yielded the CUE at *T_c_*, where *T_c_* = 25 °C.

## Results

The rate of exponential population growth (*r*) increased with selection temperature and after 10 generations, peaked at 30 °C. Growth at 33 °C was lower than predicted from the exponential relationship between temperature and population growth (Fig. 2a). However, following 100 generations of selection at 33 °C, growth increased 1.4 fold (Tukey post‐hoc test comparing long‐ and short‐term growth rates at 33 °C, *t *=* *−6.9, d.f. = 18, *P* < 0.001) to the level predicted from the initial (10 generation data) relationship between temperature and growth rate (Fig. 2a). Trajectories of specific growth rate (μ) suggest that fitness did not change over the course of the selection experiment in the ancestral lineages (20 °C) and those at 23 °C. However, between 27 and 33 °C, fitness increased over the course of 100 generations (Fig. 2b–f). The most marked response to selection (e.g. the steepest fitness trajectory) was at 33 °C (Fig. 2f; Table 1 and Table S1).

**Figure 3:**
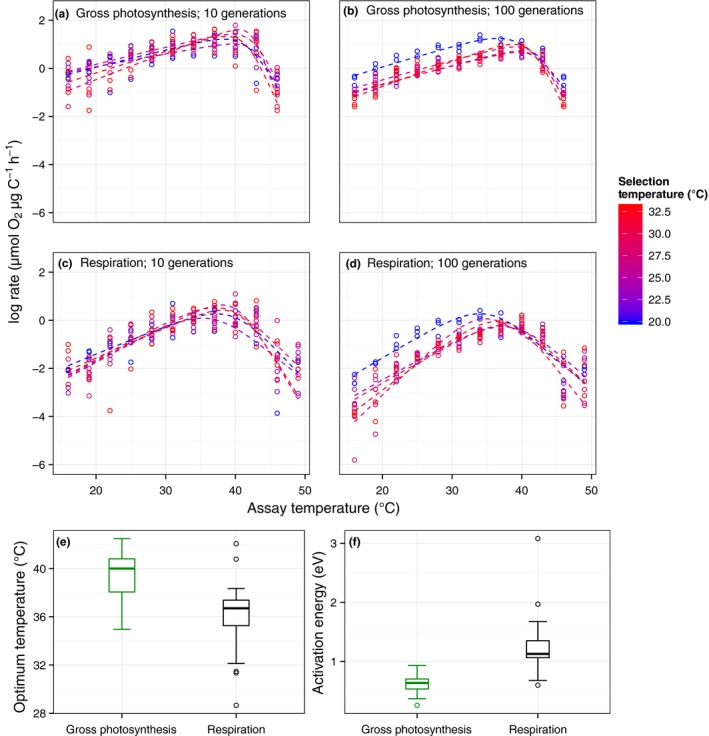
Acute effects of temperature on gross photosynthesis and respiration. Acute thermal response curves for gross photosynthesis (*P*; a, b) and respiration (*R*; c and d) were measured for populations following short‐term (10 generations, a and c) and long‐term warming (100 generations; b and d) at 20 °C (blue), through to 33 °C (red). In (a–d), fitted lines are based on mean parameters at each growth temperature across replicates (*n = *3) derived from non‐linear least squares regression using the modified Sharpe‐Schoolfield model (see eqn (1)). (e and f) Activation energies and thermal optima are pooled across all replicates and selection temperatures from both short‐ and long‐term responses because no significant effects of these variables were observed (Table 2); tops and bottoms of box‐whisker plots represent the 75th and 25th percentiles and black horizontal lines the medians.

**Figure 4:**
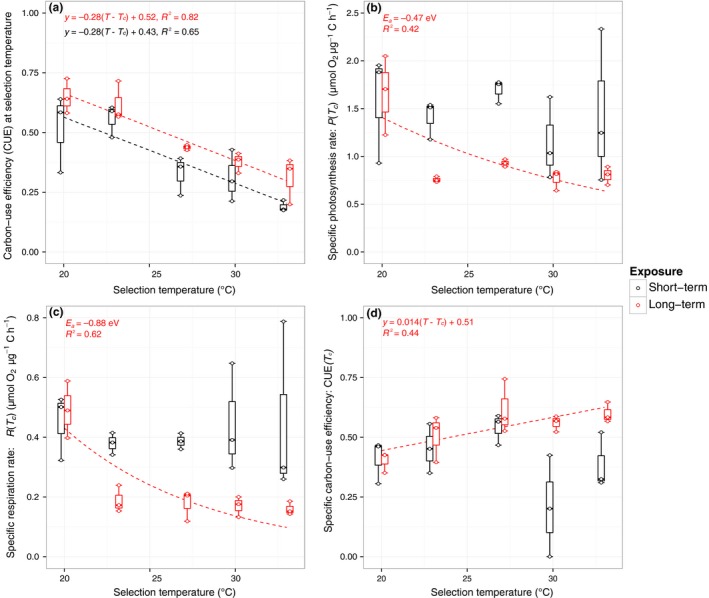
Effects of selection temperature on carbon‐use efficiency and specific rates of metabolism. (a) Carbon use efficiency (CUE) declined with increasing selection temperature after 10 (black bars) and 100 generations (red bars). However, CUE increased over the course of 100 generations (relative to the values after 10 generations), with the most marked increase at the temperature that was initially stressful (33°C). (b‐c) Specific rates of gross photosynthesis, *P*(*T_c_*) and respiration, *R*(*T_c_*) for cultures after 10 (black) and 100 (red) generations. Neither of these metabolic traits varied with selection temperature after 10 generations, but both declined exponentially following 100 generations. (d) Specific carbon‐use efficiency, CUE(*T_c_*), increased with increasing selection temperature over the course of 100 (red bars) but not after 10 generations (black bars). Fitted lines in a & d represent fits to a temperature‐centred linear regression and in b & c represent fits to the Boltzmann‐Arrhenius equation (eqn (8); see Methods).

Consistent with previous work (López‐Urrutia *et al*. 2006; Yvon‐Durocher *et al*. 2012), *R* was more sensitive to temperature than *P* in all lineages (Fig. 3a‐b; Fig. S1 & Table 2) and consequently, CUE decreased with increasing selection temperature in the lineages that had experienced both 10 and 100 generations at each temperature regime (ANCOVA *F*_1,26_ = 70.27; *P* < 0.001; Fig. 4a). Indeed, the low CUE at 33 °C was initially (after 10 generations) limiting for growth (Fig. 2a), explaining why lineages at 33 °C showed the strongest response to selection (Fig. 2f).

We hypothesised that evolution of elevated thermal tolerance should arise via increases in CUE, particularly at the temperature that was initially the most stressful (33 °C), by selection driving down *R*(*T*_c_) more than *P*(*T*_*c*_) with increasing temperature (Fig. 1d). In line with our hypothesis, CUE increased significantly between lineages exposed to 10 and 100 generations at each selection temperature (ANCOVA comparing intercepts between levels of ‘exposure’; *F*_1,26_ = 9.73; *P* = 0.004; Fig.4a). Furthermore, in the 100 generation lineages *R*(*T_c_*) and *P*(*T_c_*) declined exponentially with increasing selection temperature (Fig. 4b‐c), with the activation energy for *R*(*T_c_*) = −0.88 eV (95% CI: −1.48 to −0.46 eV), double that of *P*(*T_c_*) = −0.47 eV (95% CI: −0.82 to −0.17 eV). Consequently, the specific carbon‐use efficiency [CUE(*T_c_*) = 1−*R*(*T_c_*)/*P*(*T_c_*)] increased linearly with increasing selection temperature (ANCOVA *F*_1,13_ = 12.87; *P* = 0.003; Fig. 4d).

We found no evidence for shifts in most of the other metabolic traits – e.g. *E*_*a*_ *, T*_opt_ *, T*_*h*_ – in response to temperature, after 10 or 100 generations (Table 2; Fig. S1). Beside the documented declines in *R*(*T*_c_) and *P*(*T*_c_), the deactivation energy, *E*_*h*_, which dictates the rate at which metabolism declines past the optimum, increased with selection temperature for both *P* and *R* (Table 2; Fig. S1). Thermal optima for *P* and *R* were always higher than the selection temperatures for all lineages (Table S2). Thus, increases in *E*_*h*_, resulting in more rapid declines in metabolic rates after the optimum, would not impact growth or fitness. Decreases in specific rates of metabolism, *R*(*T*_c_) and *P*(*T*_c_), and increases in the deactivation energy, *E*_*h*_, do however suggest that metabolic thermal responses became more specialised in the lineages evolved to higher temperatures, where the strength of selection was greatest.

## Discussion

We hypothesised that the higher temperature dependence of respiration (*R*) relative to photosynthesis (*P*) would constrain growth at elevated temperatures in the model phytoplankton, *C. vulgaris*, because of reductions in carbon‐use efficiency (CUE). We therefore expected elevated thermal tolerance to evolve through down‐regulation of *R* relative to *P*, enabling more efficient allocation of fixed carbon to growth. Results from our evolution experiment were consistent with the hypotheses.

Acclimation ‐i.e. the change in physiological phenotype from a single genotype (West‐Eberhard 2003)‐ typically occurs over 1–10 generations in phytoplankton (Staehr & Birkeland 2006). In our experiment, growth rates increased exponentially between 20 and 30 °C after 10 generations, though at 33 °C, the capacity for physiological acclimation to facilitate further increases in growth was insufficient. In line with our expectations, CUE declined with increasing selection temperature, and at 33 °C growth was presumably limited by low CUE.

Evolutionary responses in phytoplankton, either via selection on pre‐existing genotype variation (Lohbeck *et al*. 2012) or *de novo* mutations, are frequently observed within 100 generations (Schaum & Collins 2014; Schlüter *et al*. 2014), and in our experiment, after 100 generations, growth at 33 °C had increased to levels predicted from the exponential relationship between temperature and growth. Consistent with our hypothesis, elevated thermal tolerance evolved via increases in CUE mediated by greater down‐regulation of specific rates of respiration, *R*(*T_c_*) relative to those of photosynthesis, *P*(*T_c_*). These findings provide direct evidence that selection on metabolic traits provides a mechanistic explanation for the evolution of elevated thermal tolerance in a model phytoplankton.

Down‐regulation of *R*(*T*_*c*_) under warming is well documented in vascular plants (Loveys *et al*. 2003; Atkin *et al*. 2015), and adjustments to *R*(*T*_*c*_), *P*(*T*_*c*_) and CUE occur within a single generation through acclimation. For plants that experience substantial variation in the environment over the course of a single generation, the capacity for physiological acclimation is likely to be key for maintaining fitness across a broad range of conditions. Phytoplankton have much shorter generation times (hours to days) and therefore the opportunity for evolutionary responses to changes in the environment, either through sorting on pre‐existing genotype variation, or *de novo* mutation and selection (Lohbeck *et al*. 2012), is much greater in absolute time. Whether mediated by acclimation or evolutionary change, the net effect of down‐regulating specific rates of respiration to facilitate growth at elevated temperatures, appears to be conserved across both vascular plants (Loveys *et al*. 2003; Atkin *et al*. 2015) and the green alga studied here. Developing a detailed understanding of the molecular and biochemical mechanisms that underpin the responses of metabolic traits to temperature should be a priority for further research.

A recent comparative analysis coupled to an eco‐evolutionary species distribution model, demonstrated that geographic variation in thermal niches of phytoplankton closely matched the temperature regime of their natal environment and suggests that thermal tolerance is a key trait shaping the response of phytoplankton communities to warming (Thomas *et al*. 2012). The rate at which thermal tolerance evolves to track changes in temperature in the Thomas *et al*. (2012) model is a key parameter for determining whether a species can persist in a given location under warming. However, due to the lack of data on rates of thermal adaptation for phytoplankton, this parameter could not be empirically constrained in the investigation by Thomas *et al*. (2012). Our experiment demonstrates that for a model species of green algae, 100 generations (45 days) was sufficient to evolve elevated rates of population growth (1.4‐fold increase) at a temperature that initially constrained growth and therefore provides an empirical basis for parameterising thermal trait evolution in eco‐evolutionary models of phytoplankton dynamics. This work also reveals the metabolic mechanisms that underpin the evolution of elevated thermal tolerance and provides a basis for refining models by linking evolution and physiology to better predict the responses of phytoplankton communities to climate change.

While our experiments focused on a single species and strain, we consider that the rapid evolution of carbon‐use efficiency will provide a mechanistic explanation for thermal adaptation in other phytoplankton. This is because the greater sensitivity of respiration to temperature than photosynthesis is well established in a wide range of autotrophs (Allen *et al*. 2005; López‐Urrutia *et al*. 2006; Staehr & Birkeland 2006; Anderson‐Teixeira *et al*. 2011; Yvon‐Durocher *et al*. 2012) and because phytoplankton have been shown to evolve rapidly in response to changes in their environment (Collins & Bell 2004; Lohbeck *et al*. 2012; Schaum & Collins 2014; Schlüter *et al*. 2014). Absolute rates of evolution will depend on generation times and genotypic variation within populations, and will intrinsically vary among species as well as being dependent on past interactions with the environment. The rates of adaptation we observe may not be consistent with those in the natural environment because experimental conditions in the laboratory are vastly simplified relative to the complex selection environment faced in nature. Our results might, for example, overestimate rates of evolution, because cultures were maintained in exponential growth. Phytoplankton in the natural environment likely spend a proportion of their life cycle at carrying capacity and/or under nutrient limitation, and thus have longer generation times than those achieved under laboratory conditions. On the other hand, our results could be conservative if the more heterogeneous environments experienced by natural populations result in high standing genetic variation. Indeed, a recent review of studies of genetic variation in natural populations using genetic markers demonstrated high standing gene and clonal diversity in diatoms, coccolithophores, dinoflagellates and a raphidopyte (Collins *et al*. 2013). Finally, rates of thermal adaptation in nature might be amplified or retarded relative to those observed in the laboratory, depending on how co‐evolutionary interactions with other species affect evolutionary responses to changes in the abiotic environment (Lawrence *et al*. 2012).

Models used to assess biogeochemical and ecological futures under climate change tend to resolve phytoplankton into either taxonomic, functional or trait‐based groups (Anderson 2005; Schneider *et al*. 2008; Vancoppenolle *et al*. 2013). More experiments are clearly needed to define the range of adaptive responses to warming among different groups of phytoplankton. Notwithstanding, our findings suggest that warm adapted phytoplankton could evolve elevated carbon fixation, which might offset some of the predicted declines in carbon sequestration under warming in aquatic ecosystems (Allen *et al*. 2005; López‐Urrutia *et al*. 2006; Yvon‐Durocher *et al*. 2010, 2012). We propose that the effects of thermal adaptation could be generalised within models based on our results; at least to test the sensitivity of model predictions to the rates of adaptation we have quantified and to challenge alternate model outputs (e.g. based on no adaptation vs. adaptation or to different rates of adaptation) with data (Stow *et al*. 2009). This will provide important insights into how the effects of thermal adaptation are expected to modify biogeochemical cycles and hence predictions of elemental fluxes and the structure and functioning food webs, under warming.
