# Supplementary material for: Rapid evolution of metabolic traits explains thermal adaptation in phytoplankton
Source: Ecol Lett. 2015 Nov 26;19(2):133–42. doi: 10.1111/ele.12545 (PMC4991271; doi:10.1111/ele.12545)
Supplement: Supplementary file 1 [file ELE-19-133-s001.pdf]

Supplementary Information

Rapid evolution of metabolic traits explains thermal adaptation in phytoplankton.

Daniel Padfield[1], Genevieve Yvon-Durocher[2], Angus Buckling[1], Simon Jennings[3,4] & Gabriel Yvon-Durocher[1*]

[1] Environment and Sustainability Institute, University of Exeter, Penryn, Cornwall, TR10 9EZ, U.K.

[2] School of Biological and Chemical Sciences, Queen Mary University of London, London E1 4NS U.K.

[3] Centre for Environmental, Fisheries and Aquaculture Science, Lowestoft, NR33 0HT, U.K.

[4] School of Environmental Sciences, University of East Anglia, Norwich, NR4 7TJ, U.K.

[*]Correspondence to: Gabriel Yvon-Durocher (g.yvon-durocher@exeter.ac.uk), Environment and Sustainability Institute, University of Exeter, Penryn, Cornwall, TR10 9EZ, U.K. TEL: 01326 259481

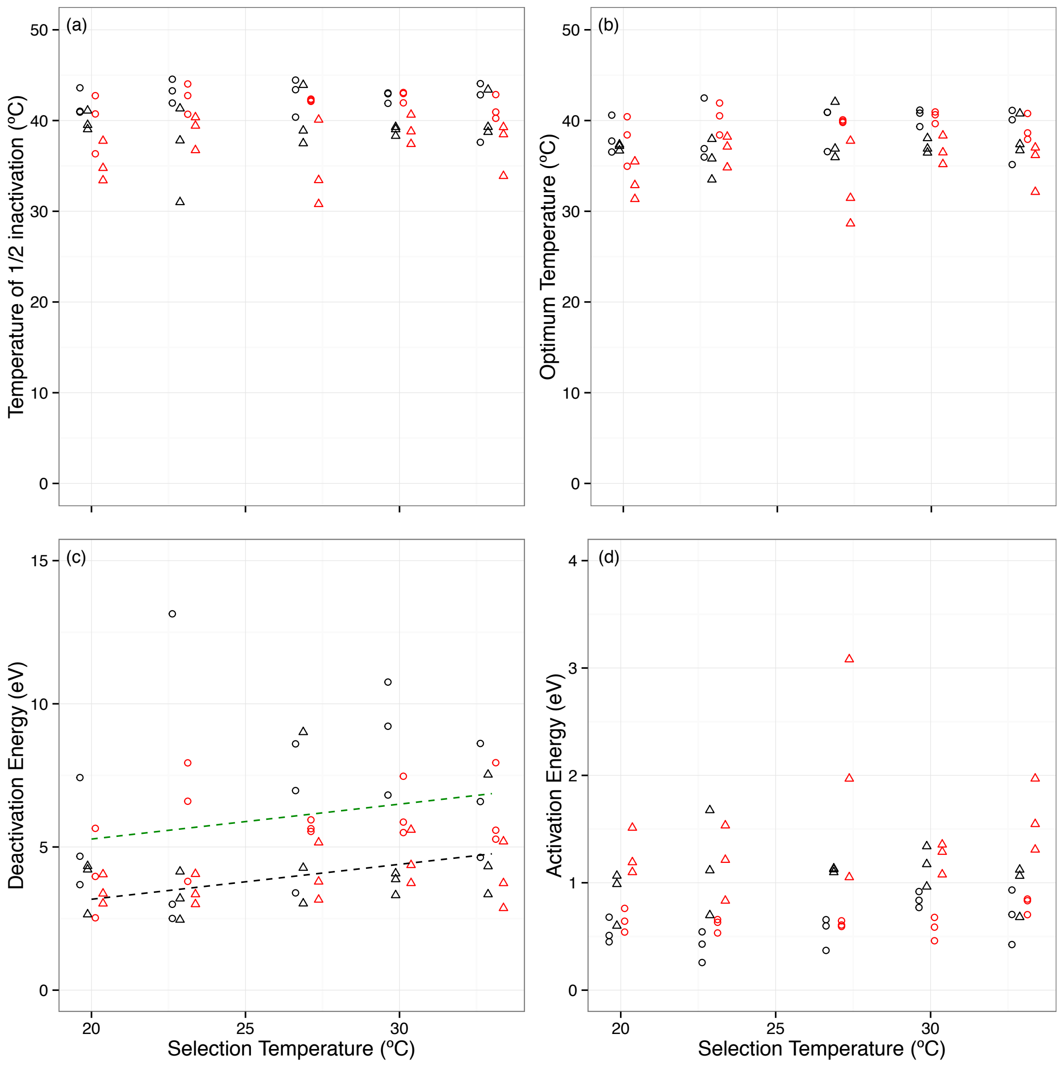

Figure S1. Effects of selection temperature on metabolic traits. In (a-d) circles show the metabolic traits of photosynthesis and triangles those of respiration after exposure to different selection temperatures for ~10 (black) and ~100 generations (red). In (c) fitted broken lines show the significant linear relationship between the deactivation energy and selection temperature for photosynthesis (green) and respiration (black) (see Table 1).

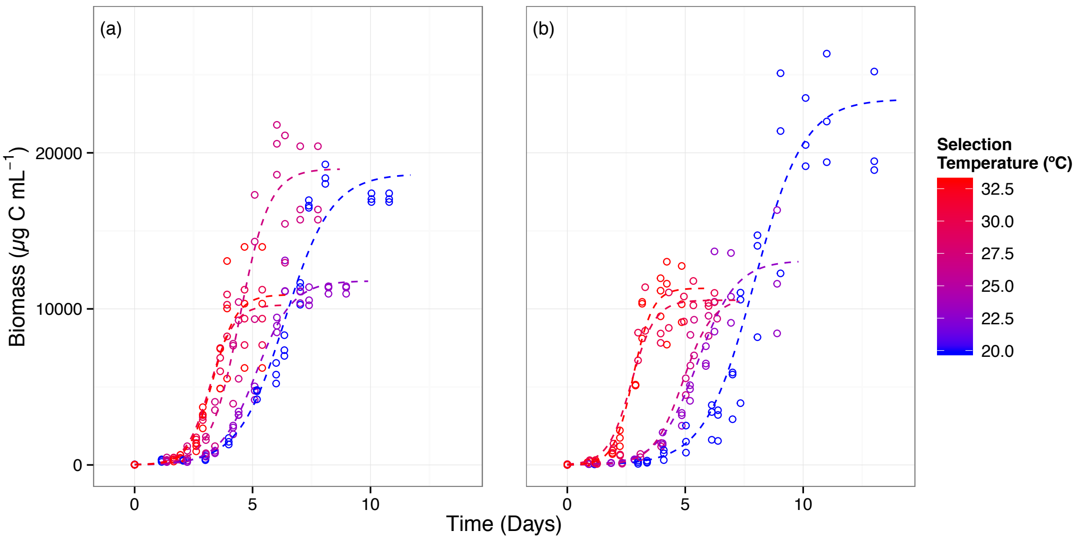

Figure S2. Effects of temperature on population dynamics. Sigmoid growth curves were measured for populations following short-term (10 generations; a) and long-term warming (100 generations; b) at 20ºC (blue), through to 33ºC (red). Fitted lines are based on mean parameters at each growth temperature from non-linear least squares regression using the sigmoid growth equation *(n = 3)* (see Methods Eq. 5).

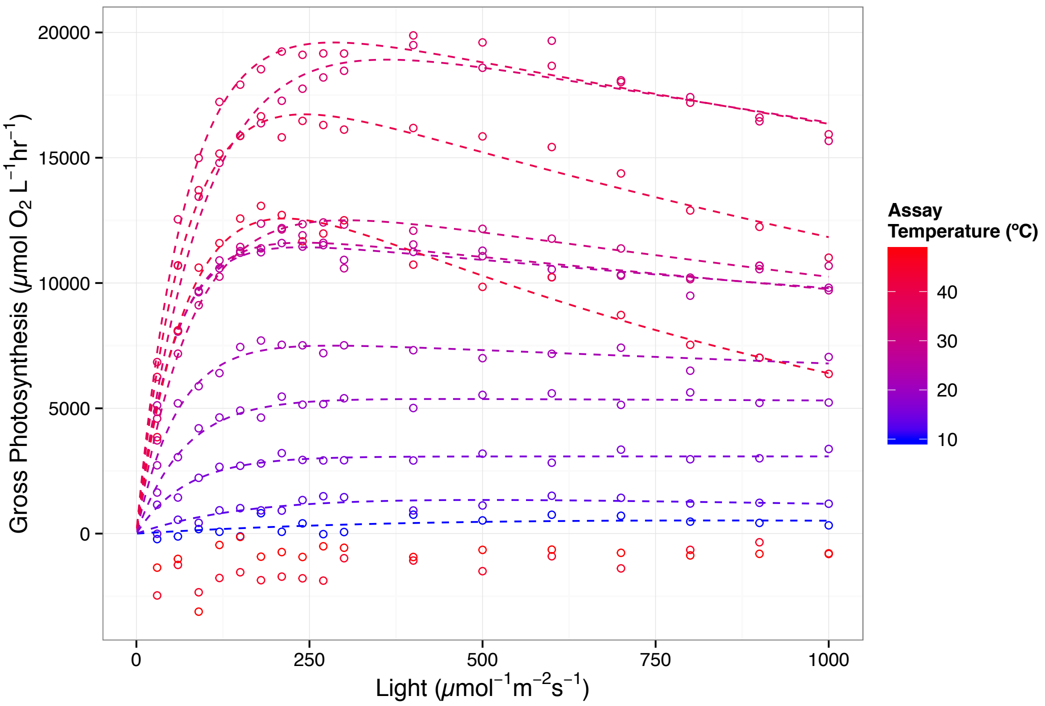

Figure S3. Photosynthesis irradiance curves used to characterise the acute temperature response of photosynthesis. Rates of gross photosynthesis (*P*) were measured at various light intensities across the full range of acute temperatures (10 – 49°C), characterising the metabolic thermal niche of *Chlorella vulgaris*. Here data are presented for one replicate at the long-term ancestral temperature regime (20ºC). Lines represent the best fit to the photoinhibition model using non-linear least squares regression (see Methods Eq. 6).

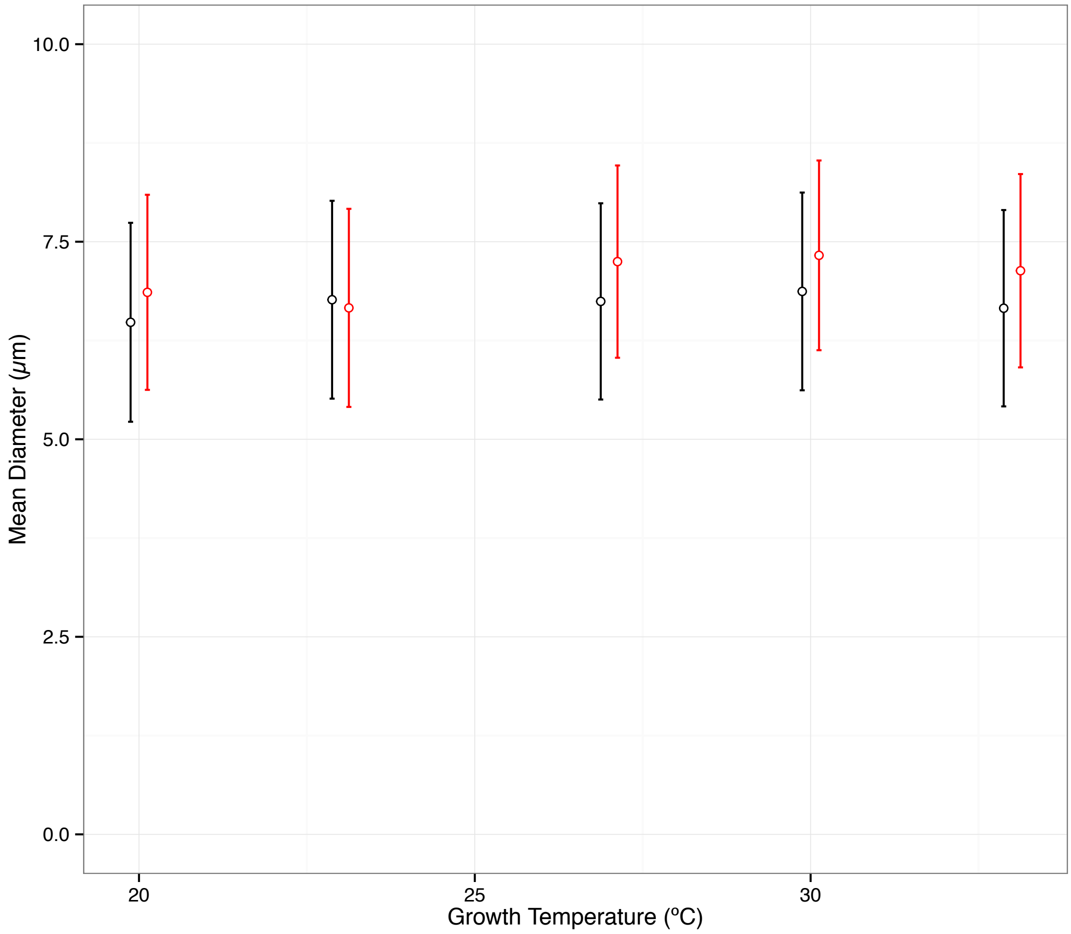

Figure S4. Effects of selection temperature on cell size. Equivalent spherical diameter was measured for each replicate after short-term (black circles) and long-term (red circles) exposure to each selection temperature. Mean cell diameter was estimated for each replicate population as the anti-log of the average $\log_{10}$ cell diameter. Body size did not vary between selection temperatures or between long-term versus short-term warming. The error bars represent 1 standard deviation from the mean.

Table S1. Trajectory of exponential growth rate at the various selection temperatures. Parameters are estimated from a mixed effects model (see Methods and Table 1) and treatment contrasts were made using Tukey's least significant difference tests. Contrasts significant at the 0.05 level for the slope of the growth trajectory are 20ºC vs 27ºC, 20ºC vs 30ºC, 20ºC vs 33ºC, 23ºC vs 27ºC, 23ºC vs 30ºC, 23ºC vs 33ºC, 27ºC vs 33ºC and 30ºC vs 33ºC. Significant contrasts for the intercept at 0.05 significance are 20ºC vs 27ºC, 20ºC vs 30ºC, 20ºC vs 33ºC, 23ºC vs 27ºC, 23ºC vs 30ºC and 23ºC vs 33ºC.

Growth Temperature	Slope (95% CI)	Intercept (95% CI)
20ºC	0.000152 (-0.000260 – 0.000566)	0.87 (0.85 – 0.89)
23ºC	0.00122 (-0.000288 – 0.00274)	1.14 (1.08 – 1.19)
27ºC	0.00452 (0.00239 – 0.00665)	1.42 (1.34 – 1.49)
30ºC	0.00556 (0.00329 – 0.00783)	1.58 (1.49 – 1.66)
33ºC	0.0130 (0. 00929 – 0.0168)	1.48 (1.36 – 1.60)

Table S2. Parameter estimates for the metabolic traits governing the thermal response curves for *Chlorella vulgaris* following fitting to a modified Sharpe-Schoolfield equation using non-linear least squares regression.

Selection Temperature	Replicate	Exposure	Flux	$b(T_c)$ (µmol O_2 µg C^{-1} h^{-1} @ 25ºC)	E_a (eV)	E_h (eV)	T_h (ºC)	T_{opt} (ºC)	R^2
20	1	short-term	R	0.5	0.99	4.33	39.04	36.69	0.46
20	2	short-term	R	0.53	1.07	4.21	39.51	37.36	0.9
20	3	short-term	R	0.32	0.6	2.64	41.09	37.19	0.58
23	1	short-term	R	0.38	0.7	3.2	41.33	37.96	0.43
23	2	short-term	R	0.41	1.68	2.46	31	33.49	0.79
23	3	short-term	R	0.34	1.12	4.14	37.8	35.81	0.78
27	1	short-term	R	0.41	1.1	3.03	37.49	35.95	0.87
27	2	short-term	R	0.36	1.12	9.01	43.92	42.06	0.76
27	3	short-term	R	0.39	1.13	4.27	38.89	36.9	0.73
30	1	short-term	R	0.3	1.34	3.31	39.04	38.05	0.86
30	2	short-term	R	0.39	1.17	4.08	38.31	36.46	0.73
30	3	short-term	R	0.65	0.96	3.87	39.28	36.9	0.88
33	1	short-term	R	0.26	1.06	3.35	39.29	37.38	0.93
33	2	short-term	R	0.3	1.12	4.32	38.74	36.72	0.18
33	3	short-term	R	0.79	0.68	7.53	43.4	40.77	0.88
20	1	short-term	P	1.88	0.51	7.42	43.61	40.6	0.91
20	2	short-term	P	1.95	0.68	4.68	40.93	37.74	0.88
20	3	short-term	P	0.93	0.45	3.68	41.03	36.54	0.51
23	1	short-term	P	1.18	0.54	13.14	44.55	42.49	0.85
23	2	short-term	P	1.51	0.26	2.5	43.27	35.97	0.54
23	3	short-term	P	1.54	0.43	3	41.93	36.9	0.69
27	1	short-term	P	1.55	0.6	3.4	40.37	36.57	0.95
27	2	short-term	P	1.75	0.66	8.6	43.4	40.92	0.87
27	3	short-term	P	1.78	0.37	6.97	44.46	40.9	0.89
30	1	short-term	P	1.03	0.84	9.22	42.95	40.82	0.91
30	2	short-term	P	0.78	0.92	10.76	43.04	41.15	0.94
30	3	short-term	P	1.62	0.77	6.81	41.9	39.33	0.91
33	1	short-term	P	0.75	0.7	6.59	42.84	40.09	0.89
33	2	short-term	P	1.25	0.93	4.64	37.61	35.15	0.56
33	3	short-term	P	2.33	0.42	8.61	44.07	41.12	0.73
20	1	long-term	R	0.4	1.19	3.02	34.77	32.88	0.6
20	2	long-term	R	0.59	1.51	3.37	33.41	31.35	0.84
20	3	long-term	R	0.49	1.1	4.04	37.76	35.49	0.76
23	1	long-term	R	0.15	1.21	4.05	39.43	37.12	0.87
23	2	long-term	R	0.24	0.83	3.35	40.36	38.21	0.95
23	3	long-term	R	0.17	1.53	3	36.73	34.83	0.92
27	1	long-term	R	0.12	3.08	3.79	30.79	28.65	0.95
27	2	long-term	R	0.2	1.97	3.16	33.43	31.47	0.92
27	3	long-term	R	0.21	1.05	5.16	40.09	37.77	0.92
30	1	long-term	R	0.2	1.29	3.74	37.41	35.19	0.75
30	2	long-term	R	0.18	1.08	5.6	40.64	38.34	0.86
30	3	long-term	R	0.13	1.36	4.37	38.8	36.49	0.9
33	1	long-term	R	0.15	1.55	5.19	38.48	36.19	0.86
33	2	long-term	R	0.19	1.31	3.74	39.27	37.02	0.84
33	3	long-term	R	0.14	1.97	2.87	33.89	32.13	0.9
20	1	long-term	P	1.22	0.54	5.65	42.74	40.42	0.82
20	2	long-term	P	2.05	0.76	2.53	36.34	34.96	0.93
20	3	long-term	P	1.7	0.64	3.97	40.73	38.42	0.94
23	1	long-term	P	0.73	0.63	6.6	42.75	40.52	0.94
23	2	long-term	P	0.79	0.66	3.8	40.7	38.41	0.96
23	3	long-term	P	0.75	0.53	7.94	44.03	41.93	0.97
27	1	long-term	P	0.93	0.61	5.95	42.37	40.08	0.91
27	2	long-term	P	0.97	0.59	5.54	42.11	39.79	0.93
27	3	long-term	P	0.89	0.65	5.64	42.23	39.92	0.98
30	1	long-term	P	0.84	0.59	7.47	43.09	40.95	0.88
30	2	long-term	P	0.81	0.46	5.5	42.98	40.64	0.83
30	3	long-term	P	0.64	0.68	5.87	41.95	39.66	0.95
33	1	long-term	P	0.7	0.83	5.58	40.94	38.64	0.95
33	2	long-term	P	0.89	0.7	7.94	42.86	40.78	0.93
33	3	long-term	P	0.81	0.85	5.27	40.25	37.93	0.86
